# Angiotensin II type 1a receptor knockout ameliorates high-fat diet-induced cardiac dysfunction by regulating glucose and lipid metabolism: AT1aR ^‒/‒^ ameliorates high-fat diet-induced cardiac dysfunction

**DOI:** 10.3724/abbs.2023054

**Published:** 2023-07-31

**Authors:** Jin Wang, Dongxue Li, Yan Zhang, Dehai Xing, Zhandong Lei, Xiangying Jiao

**Affiliations:** Key Laboratory of Cellular Physiology (Shanxi Medical University) Ministry of Education and Department of Physiology Shanxi Medical University Taiyuan 030001 China

**Keywords:** angiotensin II type 1a receptor (AT1aR), cardiac dysfunction, high-fat diet, metabolism, mitochondria

## Abstract

Obesity-related cardiovascular diseases are associated with overactivation of the renin-angiotensin system (RAS). However, the underlying mechanisms remain elusive. In this study, we investigate the role of angiotensin II (Ang II) in high-fat diet (HFD)-induced cardiac dysfunction by focusing on cardiac glucose and lipid metabolism and energy supply. Ang II plays a role in cardiovascular regulation mainly by stimulating angiotensin II type 1 receptor (AT1R), among which AT1aR is the most important subtype in regulating the function of the cardiovascular system. AT1aR gene knockout (AT1aR ^‒/‒^) rats and wild-type (WT) rats are randomly divided into four groups and fed with either a normal diet (ND) or a HFD for 12 weeks. The myocardial lipid content, Ang II level and cardiac function are then evaluated. The expressions of a number of genes involved in glucose and fatty acid oxidation and mitochondrial dynamics are measured by quantitative polymerase chain reaction and western blot analysis. Our results demonstrate that *AT1aR* knockout improves HFD-induced insulin resistance and dyslipidemia as well as lipid deposition and left ventricular dysfunction compared with WT rats fed a HFD. In addition, after feeding with HFD, AT1aR ^‒/‒^ rats not only show further improvement in glucose and fatty acid oxidation but also have a reverse effect on increased mitochondrial fission proteins. In conclusion, *AT1aR* deficiency ameliorates HFD-induced cardiac dysfunction by enhancing glucose and fatty acid oxidation, regulating mitochondrial dynamics-related protein changes, and further promoting cardiac energy supply.

## Introduction

Obesity is an independent risk factor for cardiovascular diseases (CVDs), which promotes the changes in cardiac structure and function [ 1, 2] and contributes to heart failure [3]. Despite advances in therapy, the prevalence of CVDs caused by obesity continues to increase due to its complex etiology and pathogenesis [2].

The renin-angiotensin system (RAS) is one of the most important endocrine systems [4] and plays crucial roles in regulating arterial blood pressure and blood volume. Studies in recent years have demonstrated that the RAS is involved in the occurrence and development of diabetes, hyperlipidemia, obesity and other related diseases [ 5, 6]. It has been shown that the RAS system is over-activated remarkably in obesity [ 5, 7]. As the predominant peptide of RAS, angiotensin II (Ang II) exerts cardiovascular effects mainly mediated by angiotensin II type 1 receptor (AT1R), which is closely related to myocardial hypertrophy and pathological remodeling [ 8, 9]. In rodents, there are two AT1Rs, *i.e*., AT1aR and AT1bR [10]. AT1aR, which is expressed in most tissues, is the closest homologue to human AT1R and promotes most physiological functions of Ang II, especially in cardiovascular regulation, while AT1bR is expressed prominently in adrenal and central nervous system regions [ 11‒ 13]. At present, angiotensin II receptor blockers (ARBs) have been proven to improve ventricular hypertrophy and heart failure in clinical practice [14]. However, the mechanism of RAS in cardiac dysfunction induced by obesity remains unclear and needs to be further explored.

The heart is an organ with a high demand for energy, requiring large amounts of ATP to maintain its normal systolic and diastolic functions [15]. The myocardium contains abundant mitochondria, which are the site of myocardial energy production. In order to accomplish the task of energy supply, cardiomyocyte mitochondria can convert chemical energy stored in fuel substrates (fatty acids, glucose, lactate, ketone bodies, and amino acids) into ATP through oxidative phosphorylation [ 15, 16]. Normally, cardiomyocytes obtain 70% of their energy through fatty acid oxidation, with the remaining 30% from the oxidation of substrates such as glucose and a small amount of ketone bodies and amino acids [17]. In obese patients, an imbalance between fatty acid intake and oxidation or reduced oxidative utilization of glucose may lead to disastrous consequences for cardiac function [18]. Mori *et al*. [19] revealed that an increase in Ang II could change the relationship between fatty acid oxidation and carbohydrate oxidation and significantly reduce myocardial glucose oxidation. Overexpression of angiotensinogen could also induce fatty acid oxidation, affecting the demand for myocardial energy supply [20]. Furthermore, Ang II-mediated mitochondrial respiratory enzyme damage might also lead to mitochondrial damage, resulting in reduced mitochondrial oxidative phosphorylation [21]. These studies suggested that RAS is involved in glucose and lipid metabolism in myocytes and is closely related to mitochondrial energy supply.

In the present study, we explore the role of RAS in these energy metabolism pathways after *AT1aR* gene knockout by establishing a cardiac dysfunction rat model induced by a high-fat diet (HFD), hoping to provide a novel target for the clinical treatment of cardiac dysfunction caused by obesity.

## Materials and Methods

### Experimental animals and design

Wild-type (WT) male Sprague-Dawley (SD) rats were provided by the Experimental Animal Center of Shanxi Medical University (Taiyuan, China), and *AT1aR* gene knockout (AT1aR ^‒/‒^) male SD rats were prepared by Nanjing Biomedical Research Institute of Nanjing University (Nanjing, China). Rats were all raised in specific pathogen-free (SPF) laboratory animal environmental facilities (22±2°C, 12/12 h light/dark cycle) with free access to food and water. Systemic *AT1aR*-deficient rats were generated using the sgRNA and CRISPR/Cas9 systems ( Table 1). The homozygous rats were verified by polymerase chain reaction (PCR), and the expression of *AT1aR* in major Ang II reacting tissues was determined by RT-PCR ( Supplementary Figure S1). Animal experiments were performed following the guidelines for the management of animals for medical experiments issued by the Ministry of Health of China (No. 55) and the guidelines for the management of experimental animals issued by Shanxi Medical University.

**Table:** **Table 1** The sgRNA sequences generated by AT1aR ^‒/‒^ rats generation

sgRNA name	sgRNA sequence (5′→3′)	PAM
Agtr1a-S7	GGTCTGAAGCATAGCTCGGT	TGG
Agtr1a-S8	GTGACTCAGTTACTGGTCCT	TGG
Agtr1a-S9	ATGGGCCACATACCTACGTG	TGG
Agtr1a-S10	TCTCCACTGCTAATGACTAC	AGG

Both the WT rats and AT1aR ^‒/‒^ rats were randomly divided into two groups: (1) the normal diet (ND) group fed with a normal diet; (2) the HFD group fed with a high-fat diet containing 60 kcal% fat (D12492; Research Diets, New Brunswick, USA). The four groups of experimental animals involved were 4-week-old male rats that were fed for 12 weeks, and the weights of the rats were recorded weekly. Myocardial tissue and blood samples were collected at the end of the 12th week for subsequent experiments.

### Glucose tolerance test and insulin tolerance test

Oral glucose tolerance tests (OGTTs) and insulin tolerance tests (ITTs) were performed. For OGTT, rats were fasted for 12 h. Fasting blood glucose was measured with a OneTouch Ultra glucose meter (Life Scan, Milpitas, USA), followed by glucose (2 g/kg) intragastric administration. Blood glucose levels were measured at 0, 15, 30, 60, 90 and 120 min. For ITT, rats were fasted for 6 h and then injected intraperitoneally with insulin (1 IU/kg), and blood glucose levels were assayed at 0, 15, 30, 60, 90 and 120 min after injection.

### Echocardiography and blood pressure measurement

At the end of 12 weeks of feeding, rats were anesthetized with 2%‒2.5% isoflurane, and cardiac function was assessed by transthoracic echocardiography with a GE Vivid7-Dimension Ultrasound (Wisconsin, USA). Left ventricular ejection fraction (LVEF), left ventricular fractional shortening (LVFS), left ventricular end-diastolic inner dimension (LVIDd), left ventricular end-systolic inner dimension (LVIDs), left ventricular end-diastolic volume (LVEDV), left ventricular end-systolic volume (LVESV), interventricular septal thickness at diastole (IVSd), interventricular septal thickness at systole (IVSs), left ventricular posterior wall thickness at end diastole (LVPWd) and left ventricular posterior wall thickness at end systole (LVPWs) were detected to reflect the changes of cardiac structure and function. The blood pressure was then measured by carotid intubation and monitored with a computerized BL-420 system.

### Blood lipid content assay

Plasma lipid metabolic parameters, including triglyceride (TG), total cholesterol (T-CHO), high density lipoprotein cholesterol (HDL-C), low density lipoprotein cholesterol (LDL-C) and nonesterified free fatty acids (NEFAs), were measured using commercial kits (Jiancheng Bioengineering Institute, Nanjing, China) according to the manufacturer’s instructions.

### Determination of Ang II and adenosine triphosphate (ATP) content

The Ang II concentration in the serum and heart was measured using an Ang II enzyme-linked immunoassay (ELISA) commercial kit (Bioswamp, Wuhan, China). ATP levels in myocardial tissues were measured using ATP assay kits (Jiancheng Bioengineering Institute) according to the manufacturer’s instructions.

### Hematoxylin and eosin staining

The hearts were harvested and fixed with 4% paraformaldehyde, dehydrated and embedded in paraffin, and then sliced into 4-μm sections. The heart sections were stained with hematoxylin and eosin (HE) according to standard protocol. Six fields were randomly selected to examine morphological changes in cardiomyocytes under an Olympus BX51 microscope (Tokyo, Japan).

### Oil red O staining

Heart tissues were removed and flash-frozen in liquid nitrogen, supported and embedded with optimal cutting temperature compound (OCT), and prepared into 8-μm frozen sections. Staining was carried out using the Oil red O staining kit (Solarbio, Beijing, China) according to the manufacturer’s instructions. Images were captured under an Olympus BX51 microscope at 400× magnification.

### Determination of mitochondrial respiratory chain complex V activity

The activity of mitochondrial respiratory chain complex V in myocardial tissue was determined using a mitochondrial respiratory chain complex V activity assay kit (Solarbio) according to the manufacturer’s instructions.

### Quantitative real-time polymerase chain reaction

Total mRNA was extracted from myocardial tissue using RNAiso Plus (TaKaRa, Dalian, China). Next, the concentration and purity of the extracted mRNA were measured using NANODROP ONE (Thermo Scientific, Waltham, USA). Total RNA was then reverse-transcribed into cDNA using the PrimeScript RT kit (TaKaRa). PCR was performed on a LightCycler 96 real-time PCR system (Roche, Basel, Switzerland) using TB Green Primers Ex *Taq* II (TaKaRa). The sequences of primers involved are shown in Table 2. The 2 ^‒ΔΔCT^ method was used to calculate the relative gene expression. *β-Actin* was used as an internal reference.

**Table:** **Table 2** Primer sequences for quantitative real-time PCR

Gene	Forward primer (5′→3′)	Reverse primer (5′→3′)
*Agtr1a*	CCCACTCAAGCCTGTCTACGAA	GTGTGCTTTGAACCTGTCACTCC
*HK2*	GACAATGGCTGCCTGGATGA	TCCCAAGTACATGCCGCTGA
*PFK1*	TGAATGCTGCAGTTCGCTCTAC	ACATAGCTCCAGCCAGCTTCC
*PK*	TCGTGTGAACTTGGCCATGAA	CATGGTGTTGGTGAAGCCAGA
*CD36*	AACCCAATGGAGCCATCTTTGA	GTTGAGCACACCTTGAACAAATGAG
*PPARα*	GGCAATGCACTGAACATCGAG	GCCGAATAGTTCGCCGAAAG
*PPARδ*	ACTGCAGCTGCCTTGGTGTAA	ACCTGCTCACAGACCATCAATTC
*PPARγ*	TGGAGCCTAAGTTTGAGTTTGCTG	GATGTCCTCGATGGGCTTCAC
*PGC-1α*	GCACTGACAGATGGAGACGTGA	TCATTGTAGCTGAGCTGAGTGTTGG
*ACOX1*	GGCCGCTATGATGGAAATGTG	GGGCTTCAAGTGCTTGTGGTAA
*ACADM*	GCGTGACAGAACCCTCAGCA	TCCACGATGAATCCGGTGAA
*β-actin*	ACGGTCAGGTCATCACTATCG	GGCATAGAGGTCTTTACGGATG

### Western blot analysis

The heart tissues were homogenized with a tissue grinder at a low temperature. Then, the lysate was centrifuged at 9660 *g* for 20 min, and the supernatant was retained to obtain total protein. The protein concentration was determined using a BCA kit (Boster, Wuhan, China). After denaturation, the protein sample (20 μg) was separated by 10% sodium dodecyl sulphate-polyacrylamide gel electrophoresis (SDS-PAGE) and then transferred to polyvinylidene difluoride (PVDF) membranes. Then, the membranes were blocked with 5% skim milk powder or bovine serum albumin (BSA) for 2 h, washed with TBST, and incubated with primary antibodies at 4°C overnight. Specific primary antibodies were as follows: anti-AT2R (Abcam, Cambridge, UK), anti-CPT1B (Abclonal, Wuhan, China), anti-CD36 (Abclonal), anti-P-PDH (CST, Beverly, USA), anti-PDH (CST), anti-MFN2 (Abclonal), anti-DRP1 (Abclonal), anti-FIS1 (Abclonal) and anti-β-actin (Bioworld, Minneapolis, USA). The membranes were washed and incubated with the horseradish peroxidase-labelled secondary antibody (Boster) at room temperature for 1 h. Finally, the bands were visualized using an ultrasensitive enhanced chemiluminescence (ECL) detection kit (SEVEN BIOTECH, Corp., Ltd., Beijing, China) and exposed with a ChemiDoc Touch Imaging System (Bio-Rad, Hercules, USA). The images were quantitatively analyzed by densitometry using ImageJ software, and β-actin was used as the loading control.

### Statistical analysis

Data were analyzed by GraphPad Prism8 software (GraphPad Software, Inc., San Diego, USA) and expressed as the mean± standard error of the mean (SEM). One-way analysis of variance (ANOVA) was used to compare the differences between groups. *P*<0.05 was considered statistically significant.

## Results

### *AT1aR* knockout improved metabolic disorder and hypertension in rats fed with HFD

**Figure 1:**
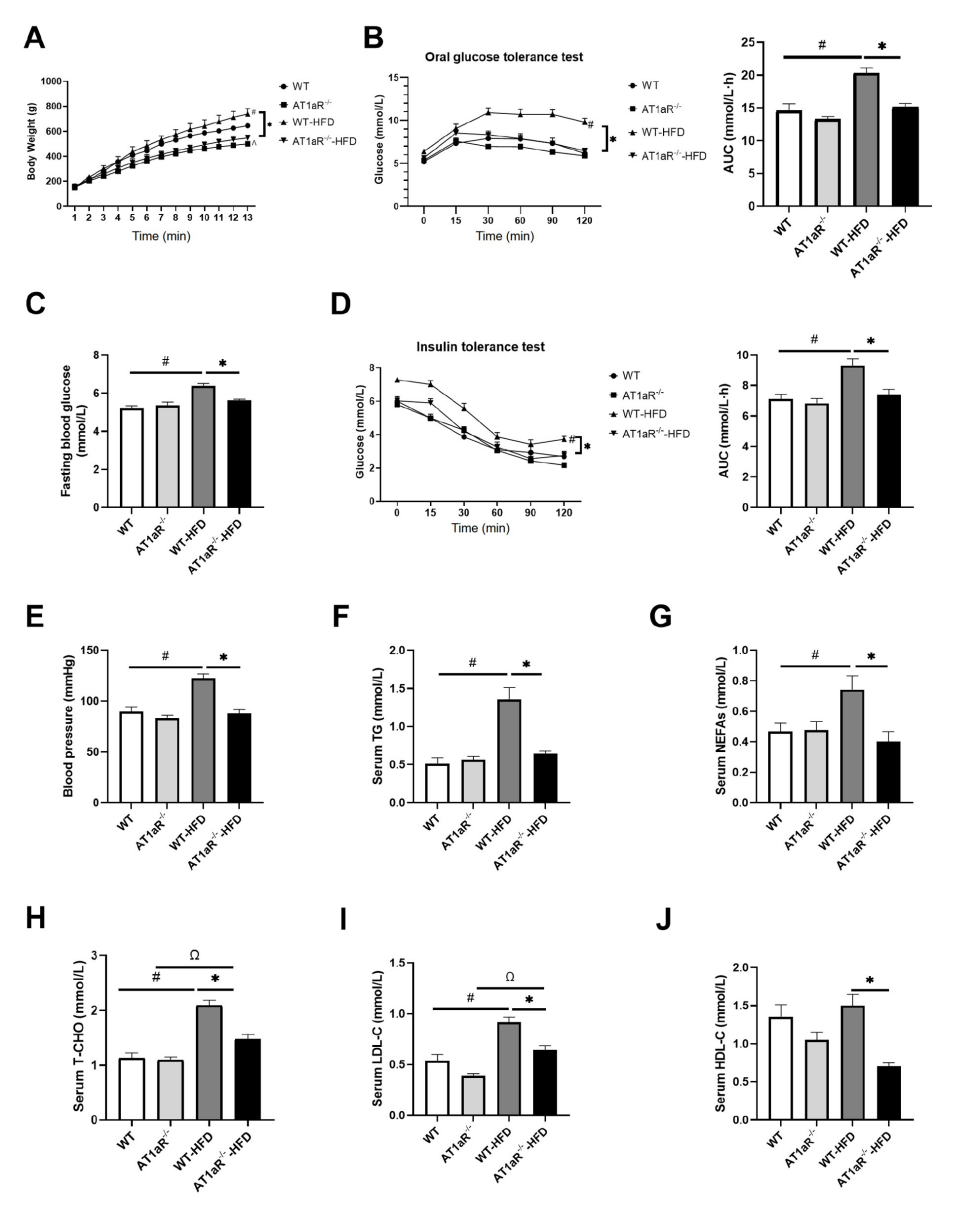
Figure 1 *AT1aR* knockout improved metabolic disorder and hypertension in rats fed with HFD (A) The weight of the rats was recorded weekly. (B) Oral glucose tolerance test (OGTT) and area under the curve (AUC). (C) Fasting blood glucose (FBG). (D) Insulin glucose tolerance test (ITT) and area under the curve (AUC). (E) Blood pressure measurement. (F) Serum triglyceride (TG) levels. (G) Serum free fatty acids (NEFAs). (H) Serum total cholesterol (T-CHO). (I) Serum low-density lipoprotein cholesterol (LDL-C). (J) Serum high-density lipoprotein cholesterol (HDL-C). n=5. Data are shown as the mean±SEM. * P<0.05, AT1aR‒/‒-HFD vs WT-HFD rats; # P<0.05, WT vs WT-HFD rats; ΩP<0.05, AT1aR‒/‒ vs AT1aR‒/‒-HFD rats; ΛP <0.05, WT vs AT1aR‒/‒ rats. AT1aR, angiotensin II type 1a receptor; WT, wild type.

The weight changes were recorded weekly during feeding. The body weight of WT rats fed with HFD was significantly increased, while the body weight of AT1aR ^‒/‒^ rats fed with HFD was markedly lower than that of WT rats fed with HFD ( Figure 1A). Compared to WT rats fed with HFD, *AT1aR* knockout significantly decreased the level of fasting blood glucose ( Figure 1C), and markedly elevated glucose tolerance and insulin sensitivity, as detected by OGTT and ITT respectively ( Figure 1B,D). Meanwhile, high-fat diet induced hypertension in WT rats, which was attenuated by *AT1aR* deficiency ( Figure 1 E).

Furthermore, the TG, NEFAs, T-CHO, LDL-C and HDL-C in WT rats fed with HFD were significantly higher than those in WT rats fed with ND, whereas these changes were obviously reversed in AT1aR ^‒/‒^ rats fed with HFD ( Figure 1F‒J). These results demonstrated that *AT1aR* gene knockout improved insulin sensitivity and alleviated hypertension and lipid metabolism disorder induced by HFD feeding.

### AT1aR deletion ameliorated cardiac dysfunction, cardiomyocyte hypertrophy and lipid deposition induced by HFD

**Figure 2:**
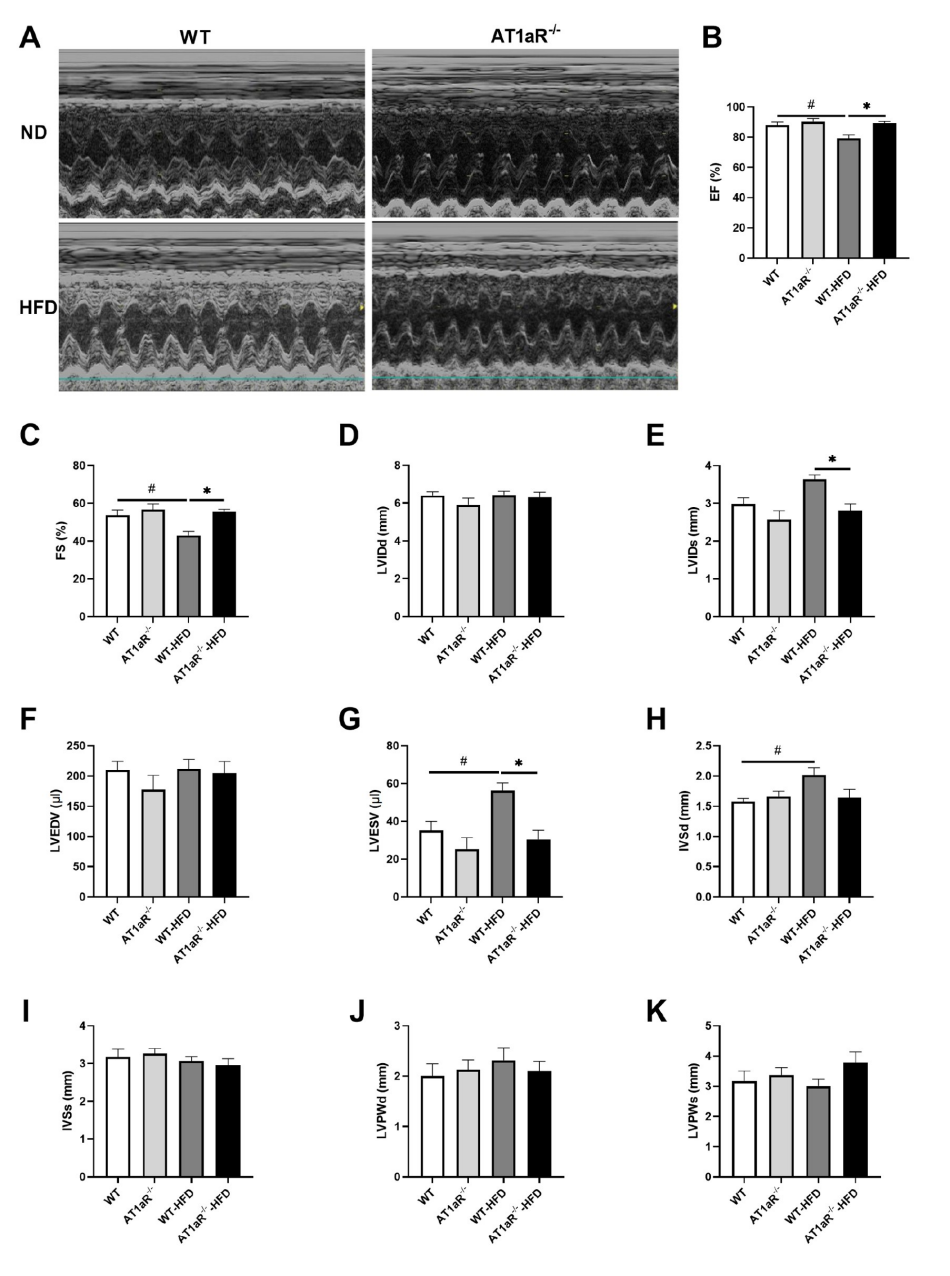
Figure 2 *AT1aR* knockout ameliorated left ventricular dysfunction induced by HFD in rats (A) Representative transthoracic echocardiographic images of each group after 12 weeks of feeding. (B‒K) Statistical analysis of echocardiography EF, FS, LVIDd, LVIDs, LVEDV, LVESV, IVSd, IVSs, LVPWd and LVPWs. n=5. Data are shown as the mean±SEM. *P<0.05, AT1aR‒/‒-HFD vs WT-HFD rats; #P<0.05, WT vs WT-HFD rats. EF, ejection fraction; FS, fractional shortening; LVIDd, left ventricular end-diastolic inner dimension; LVIDs, left ventricular end-systolic inner dimension; LVEDV, left ventricular end-diastolic volume; LVESV, left ventricular end-systolic volume; IVSd, interventricular septal thickness at diastole; IVSs, interventricular septal thickness at systole; LVPWd, left ventricular posterior wall thickness at end diastole; and LVPWs, left ventricular posterior wall thickness at end systole.

Left ventricular dimensions and function were assessed by transthoracic echocardiography ( Figure 2A‒H). LVEF and LVFS reflect left ventricular systolic function, while LVESV, LVIDs, IVSd, LVPWd and LVPWs reflect cardiac morphology. There was no significant difference in cardiac ultrasound indexes between WT rats and AT1aR ^‒/‒^ rats fed with ND. Compared with WT rats fed with ND, the LVEF and LVFS in WT rats fed with HFD were decreased, while the LVESV and IVSd values were increased ( Figure 2B,C,G,H). In contrast, the LVEF and LVFS of AT1aR ^‒/‒^ rats fed with HFD were higher than those of WT rats fed with HFD, while LVIDs and LVESV were lower ( Figure 2B,C,E,G). No significant differences were observed in LVIDd, LVEDV, IVSs, LVPWd and LVPWs between WT and AT1aR ^‒/‒^ rats fed with HFD. These results showed that *AT1aR* knockout ameliorated cardiac dysfunction caused by HFD feeding.

**Figure 3:**
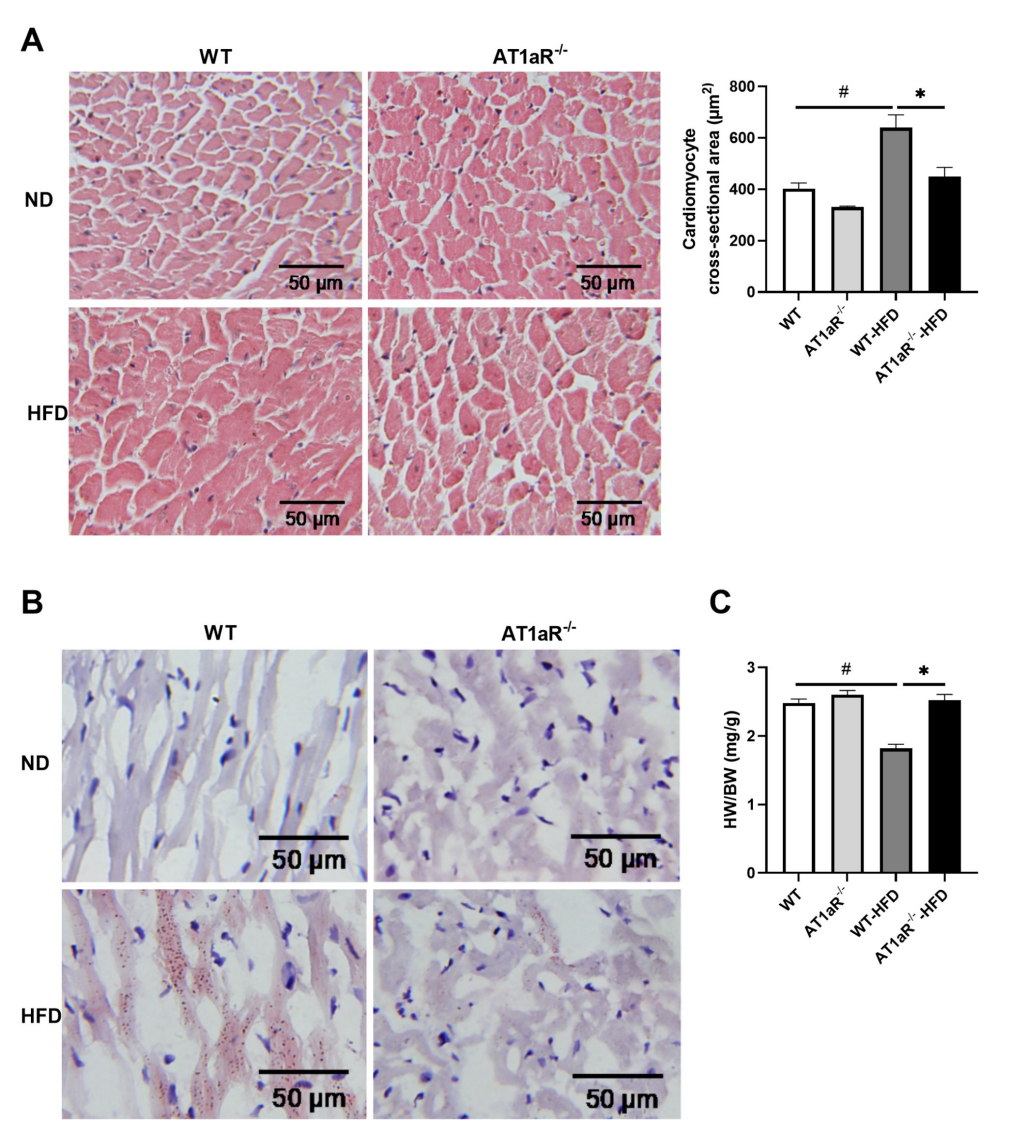
Figure 3 *AT1aR* knockout alleviated HFD-induced cardiomyocyte hypertrophy and lipid deposition (A) HE staining showed the morphology of cardiomyocytes. ImageJ was used to calculate the cross-sectional area of cardiomyocytes. Scale bar: 50 μm, n=3. (B) Oil red O staining showed lipid droplet deposition in cardiomyocytes. Scale bar: 50 μm. n=5. (C) The heart weight (HW) to body weight (BW) ratio was determined. n=5. Data are shown as the mean±SEM. * P<0.05, AT1aR‒/‒-HFD vs WT-HFD rats; # P<0.05, WT vs WT-HFD rats.

Next, paraffin sections of hearts were stained with hematoxylin-eosin (HE) to show the histomorphology of cardiomyocytes ( Figure 3A). Significant cardiomyocyte hypertrophy was observed in WT rats fed with HFD but not in AT1aR ^‒/‒^ rats. Then, Oil red O staining was employed to observe the distribution of lipid droplets in myocardial tissue ( Figure 3B). There were fewer lipid droplets in the cardiomyocytes of WT and AT1aR ^‒/‒^ rats in the ND group. After HFD feeding, there were a large number of fine lipid droplets in the cardiomyocytes of WT rats, which were distributed along the myocardial fibers, while the lipid droplets in the cardiomyocytes of AT1aR ^‒/‒^ rats fed with HFD were significantly reduced.

Furthermore, the relative changes of heart hypertrophy and body weight gain were analyzed by measuring the heart weight to body weight ratio (HW/BW) in rats. There was no significant difference in the ratio between WT and AT1aR ^‒/‒^ rats in the ND group. The ratio of WT rats fed with HFD was decreased obviously, whereas the change was reversed in AT1aR ^‒/‒^ rats fed with HFD ( Figure 3C). These results suggested that *AT1aR* knockout attenuated HFD-induced cardiomyocyte hypertrophy and lipid deposition.

### *AT1aR* deficiency alleviated adverse effects on cardiac energy supply after HFD feeding

**Figure 4:**
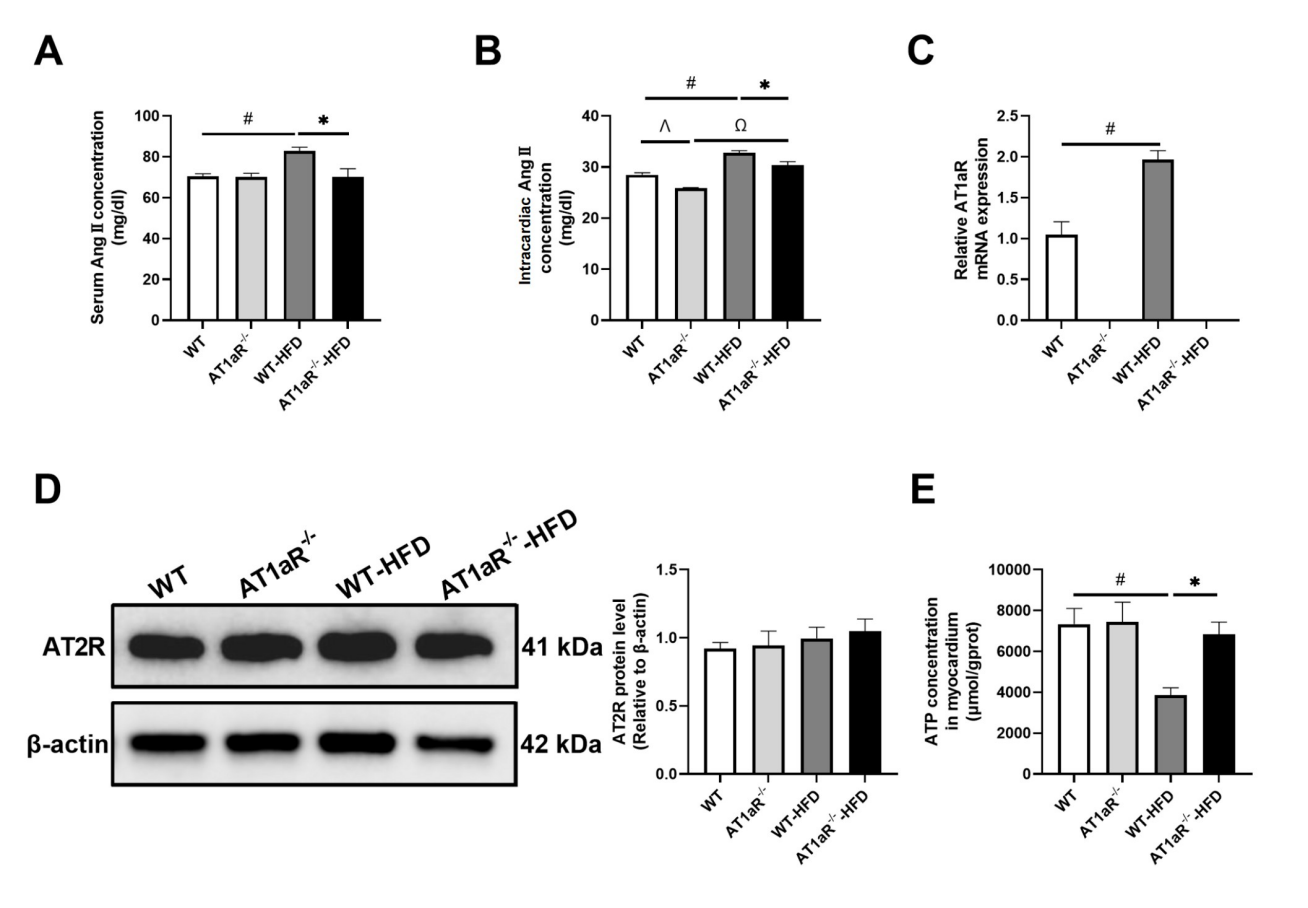
Figure 4 *AT1aR* deficiency alleviated adverse effects on cardiac energy supply after HFD feeding (A,B) The content of angiotensin II in the serum and heart was determined. (C) The mRNA expression of AT1aR in the heart was detected by real-time PCR. (D) The protein expression of AT2R was detected by western blot analysis. (E) Adenosine triphosphate (ATP) levels in the heart. n=5. Data are shown as the mean±SEM. *P<0.05, AT1aR‒/‒-HFD vs WT-HFD rats; #P<0.05, WT vs WT-HFD rats; Ω P<0.05, AT1aR‒/‒ vs AT1aR‒/‒-HFD rats; Λ P<0.05, WT vs AT1aR‒/‒ rats.

Ang II content in serum and myocardium was measured to reflect the activity of the RAS system in rats. Compared with WT rats fed with ND, Ang II content in the serum and myocardium of WT rats fed with HFD was increased obviously ( Figure 4A,B). However, the Ang II level in AT1aR ^‒/‒^ rats fed with HFD was lower than that in the HFD-fed WT rats. Correspondingly, the gene expression of AT1aR in WT rats after HFD feeding was higher than that in the ND group ( Figure 4C). Meanwhile, the AT2R protein expression level did not change significantly in all groups ( Figure 4D). These results indicated the activation of the RAS system and the corresponding AT1aR in rats in response to HFD.

In addition, the myocardial ATP level was measured to reflect the energy supply of the heart. The ATP content in WT rats fed with HFD was significantly decreased compared with that in WT rats fed with ND, while the ATP level in AT1aR ^‒/‒^ rats showed little change after HFD feeding ( Figure 4E). Therefore, the myocardial ATP content in AT1aR ^‒/‒^ rats after HFD feeding was higher than that in WT rats fed with HFD. These results suggested that *AT1aR* deficiency alleviated adverse effects on cardiac energy supply and inhibited the RAS system.

### *AT1aR* deficiency improved the decreased glucose oxidation function after HFD feeding

**Figure 5:**
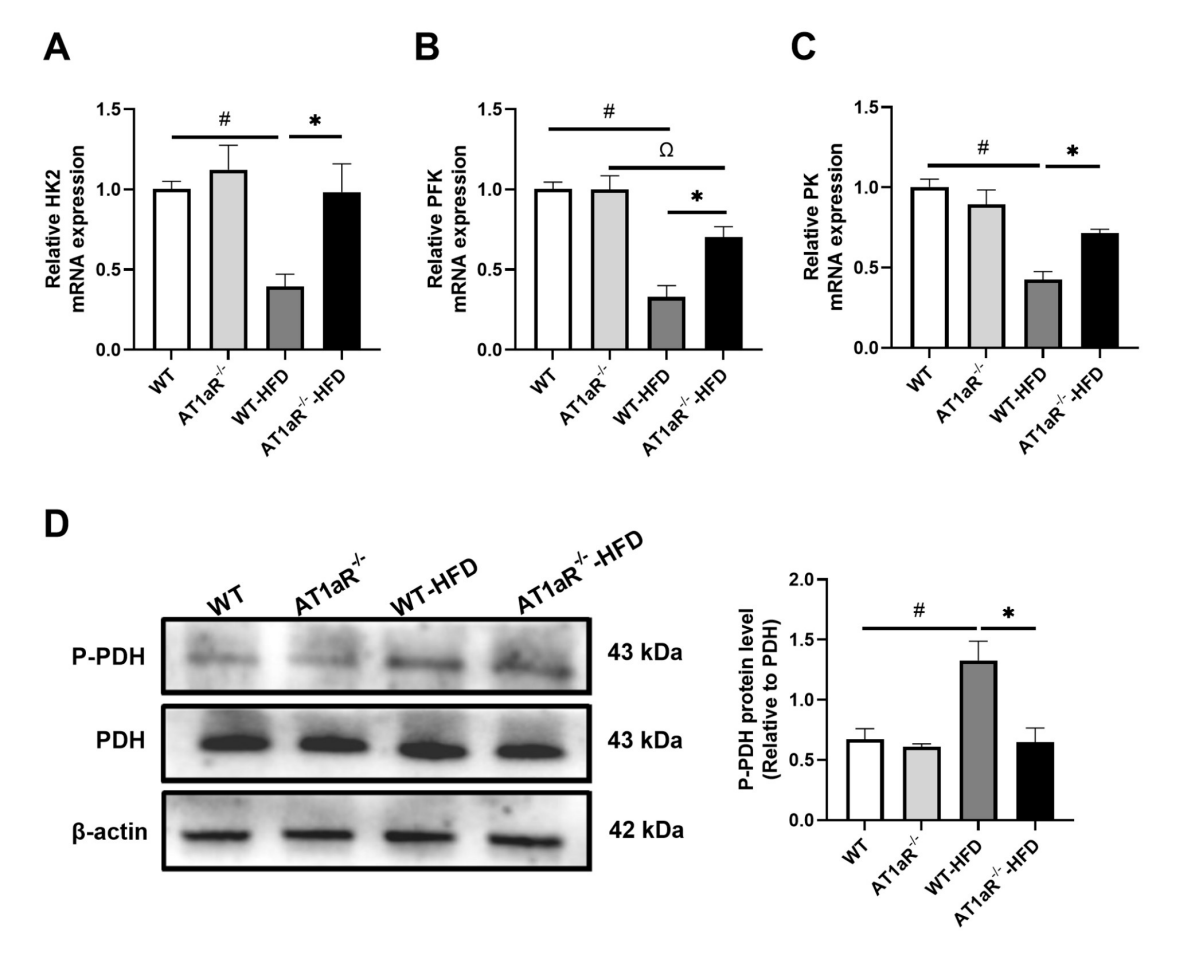
Figure 5 *AT1aR* deficiency improved the decreased glucose oxidation function after HFD feeding (A‒C) The mRNA expressions of HK2, PFK and PK in the heart were detected by real-time PCR. (D) The protein expressions of P-PDH and PDH in each group were detected by western blot analysis. n=4. Data are shown as the mean±SEM. *P<0.05, AT1aR‒/‒-HFD vs WT-HFD rats; #P<0.05, WT vs WT-HFD rats; ΩP<0.05, AT1aR‒/‒ vs AT1aR‒/‒-HFD rats. HK2, hexokinase 2; PFK, phosphofructokinase; PK, pyruvate kinase; P-PDH, phosphorylated pyruvate dehydrogenase; PDH, pyruvate dehydrogenase.

Pyruvate enters the mitochondria to initiate the aerobic oxidation of glucose, so the key enzymes that affect pyruvate production during glycolysis also play a crucial role in the subsequent oxidation process. To explore the effect of *AT1aR* deficiency on glucose oxidation after HFD feeding, we determined the gene expression levels of the key glycolytic enzymes hexokinase (HK), phosphofructokinase 1 (PFK1) and pyruvate kinase (PK) in cardiomyocytes. The gene expression levels of HK2, PFK1 and PK in WT rats fed with HFD were decreased compared with those in WT rats fed with ND, while *AT1aR* knockout attenuated this adverse effect ( Figure 5A‒C). Pyruvate dehydrogenase (PDH) is a key regulator of pyruvate entering mitochondria for glucose oxidation, and we detected the protein expression level of PDH to reflect this oxidation function. The phosphorylated PDH (P-PDH) level in WT rats fed with HFD was higher than that in WT rats fed with ND, while the protein level of P-PDH in AT1aR ^‒/‒^ rats fed with HFD was lower than that in WT rats fed with HFD ( Figure 5D). The results suggested that *AT1aR* knockout may increase cardiac energy supply after HFD feeding by improving glucose oxidation function.

### *AT1aR* knockout promoted decreased fatty acid oxidation after HFD feeding

**Figure 6:**
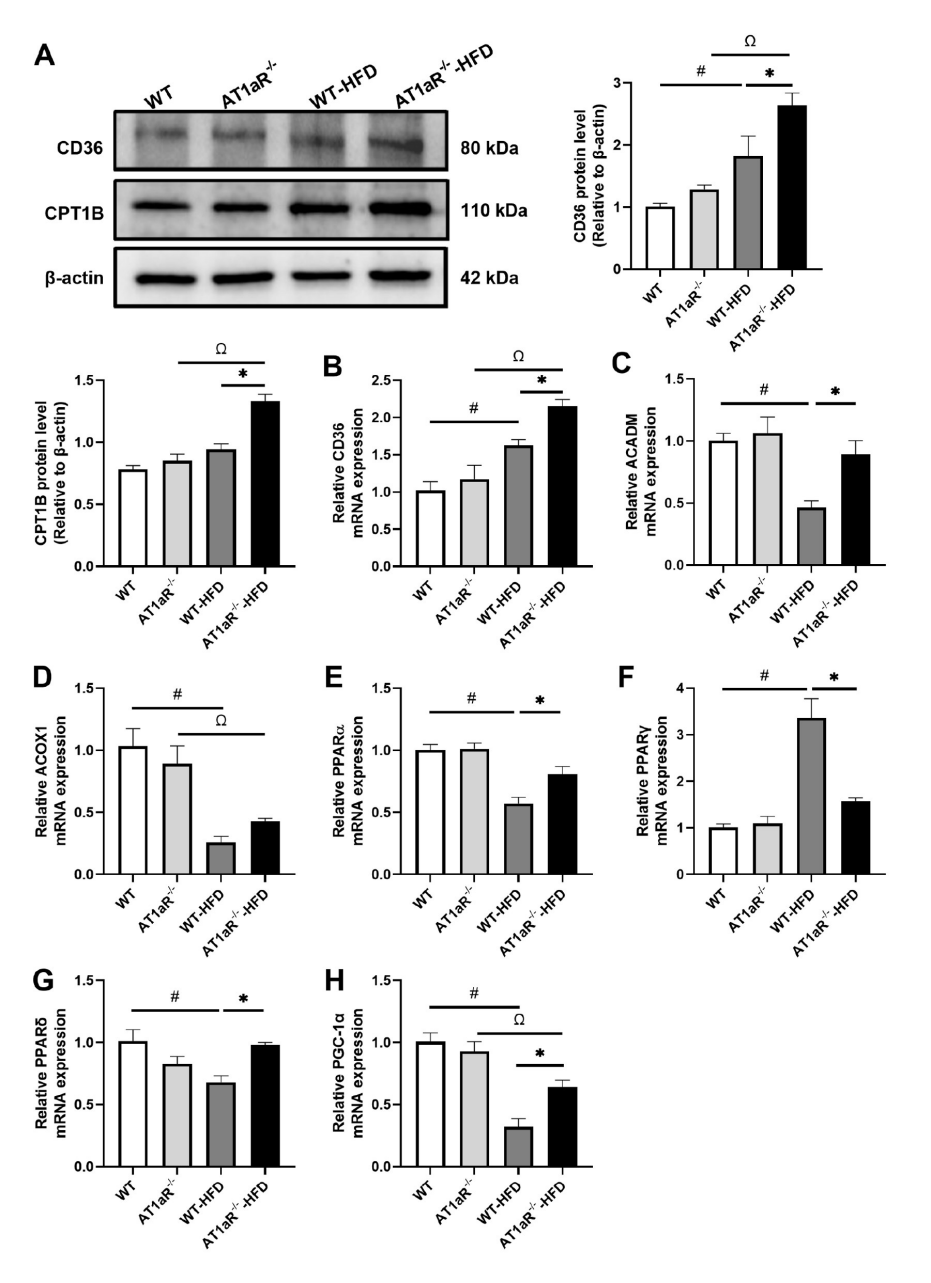
Figure 6 *AT1aR* knockout promoted decreased fatty acid oxidation after HFD feeding (A) The protein expressions of CD36 and CPT1B in each group were detected by western blot analysis. (B‒H) The mRNA expressions of CD36, ACADM , ACOX1, PPARα, PPARγ, PPARδ and PGC-1α in the heart were detected by real-time PCR. n=4. Data are shown as the mean±SEM. *P<0.05, AT1aR‒/‒-HFD vs WT-HFD rats; #P<0.05, WT vs WT-HFD rats; ΩP<0.05, AT1aR‒/‒ vs AT1aR‒/‒-HFD rats. CD36, fatty acid translocase; CPT1B, carnitine palmitoyl transferase 1B; PPARα/γ/δ, peroxisome proliferator-activated receptor alpha/gamma/delta; PGC-1α, PPARγ coactivator-1α.

Free fatty acids are transported into cardiomyocytes mainly by fatty acid translocase (FAT/CD36) for oxidative utilization [22]. Therefore, the expression levels of the CD36 gene and protein can reflect the uptake capacity of myocardial fatty acids. Carnitine palmitoyl transferase 1 (CPT1) is a rate-limiting enzyme of fatty acid uptake and β-oxidation in mitochondria. It can convert fatty acyl-CoA into the corresponding acylcarnitine in the outer membrane of mitochondria. In terms of protein levels, the expression levels of CD36 and CPT1B in AT1aR ^‒/‒^ rats in the HFD group were higher than those in WT rats in the HFD group, and the CD36 gene expression level also supported this result ( Figure 6A,B). The above results indicated that fatty acid intake was increased in rats after HFD feeding, while AT1aR ^‒/‒^rats fed with HFD showed a more significant increase.

Fatty acid oxidation-associated enzymes also include acyl-coenzyme A oxidase 1 (ACOX1) and acyl-coenzyme A dehydrogenase (ACADM), as well as the peroxisome proliferator-activated receptor (PPAR) family, which are responsible for upstream regulation. There are three members of the PPAR family, PPARα, PPARβ/δ and PPARγ, with different but overlapping regulatory expression patterns [23]. PPARα, as a nuclear receptor transcription factor, regulates β-oxidation of cardiac fatty acids, depending on peroxisome and mitochondrial enzyme activities. PPARβ/δ is also involved in the regulation of fatty acid oxidation in the heart and skeletal muscle, while PPARγ plays a role in adipose differentiation and storage. PPARγ coactivator-1α (PGC-1α) is highly expressed in cardiomyocytes and is a key factor in the regulation of myocardial mitochondrial biogenesis [24], which is coregulated with PPARα. In this study, we found that the gene expressions of *ACADM*, *ACOX1* , *PPARα*, *PPARβ*/ *δ* and *PGC-1α* were decreased and *PPARγ* was increased in HFD-fed WT rats compared with ND-fed WT rats. However, *AT1aR* knockout reversed these alterations caused by HFD feeding ( Figure 6C‒H). These results confirmed that *AT1aR* deficiency enhanced the impaired fatty acid oxidation capacity after HFD feeding, both in terms of upstream regulation level and downstream oxidation utilization.

### Deletion of *AT1aR* affected the changes in mitochondrial dynamics

**Figure 7:**
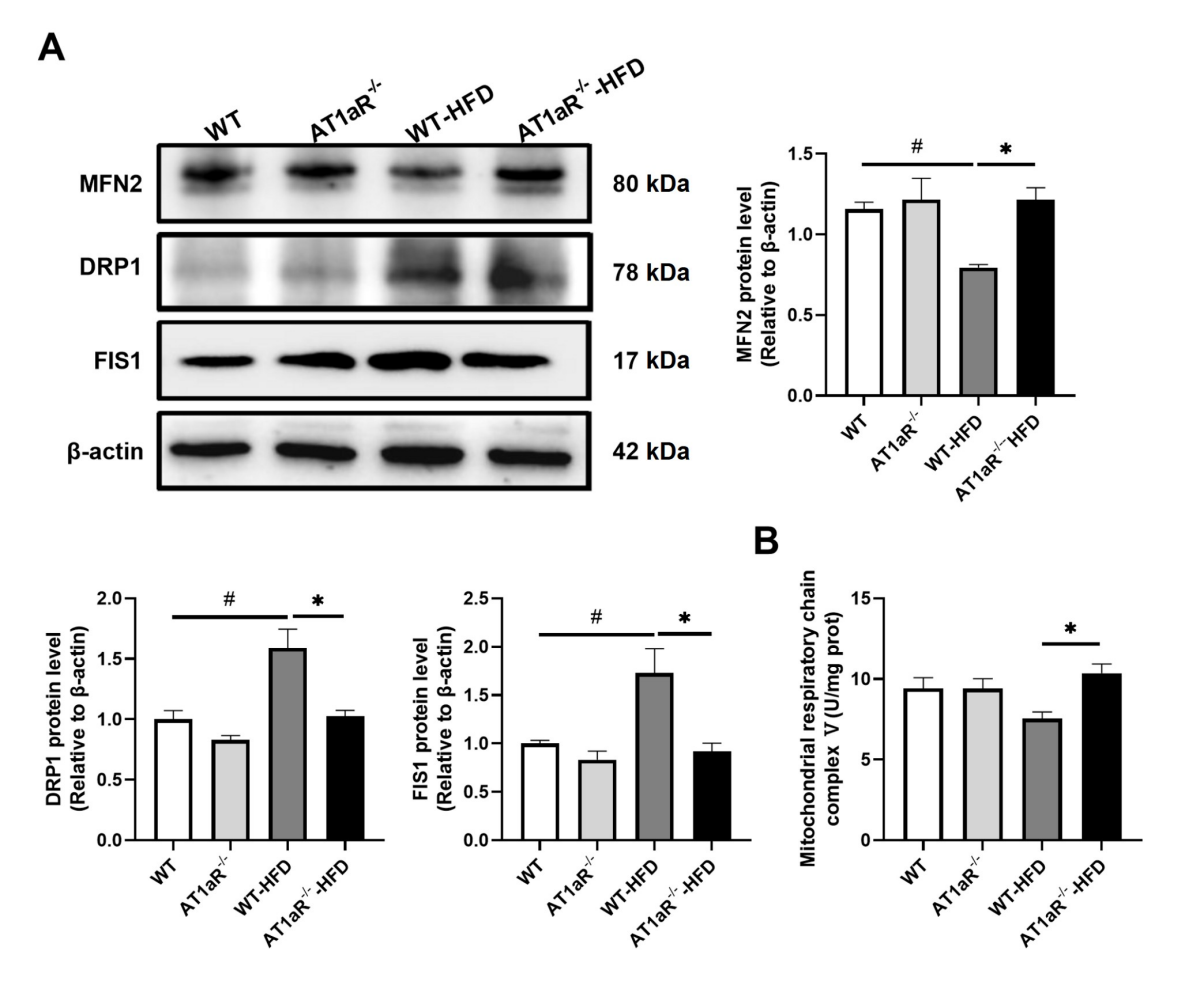
Figure 7 Deletion of *AT1aR* affected the changes in mitochondrial dynamics (A) The protein expressions of MFN2, DRP1 and FIS1 in each group were detected by western blot analysis. (B) Mitochondrial respiratory chain complex enzyme V activity was measured in each group. n=4. Data are shown as the mean±SEM. * P<0.05, AT1aR‒/‒-HFD vs WT-HFD rats; # P<0.05, WT vs WT-HFD rats. MFN2, mitochondrial fusion protein 2; DRP1, dynamin-related peptide 1; FIS1, mitochondrial fission protein 1.

Maintaining mitochondrial function and integrity is essential for the heart, an organ with high energy requirements. Mitochondrial dynamics depends on the counterbalance between mitochondrial fission and fusion [25]. The functional status of mitochondria was further evaluated by measuring the protein expression levels of mitochondrial fission and fusion proteins. Compared with WT rats fed with ND, WT rats fed with HFD showed decreased mitochondrial fusion protein 2 (MFN2) but increased mitochondrial fission 1 protein (FIS1) and dynamin-related protein 1 (DRP1). Then, changes in mitochondrial fusion and fission proteins were reversed by *AT1aR* gene knockout ( Figure 7A).

In addition, we also examined the activity of mitochondrial respiratory chain complex V (ATP synthase) to reflect the function of mitochondrial oxidative phosphorylation to produce ATP. It was obvious that the level of complex V in AT1aR ^‒/‒^ rats fed with HFD was higher than that in WT rats in the HFD group ( Figure 7B). These results indicated that *AT1aR* knockout alleviated the increase in mitochondrial fission caused by HFD feeding and improved mitochondrial function.

## Discussion

The prevalence of obesity and obesity-related diseases is growing globally [26], which increases the cardiovascular morbidity and mortality of the population and poses a great threat to public health [27]. In addition, overactivation of RAS is a common event in obese subjects [ 15, 28]. Ang II is the major effector of RAS that plays a crucial role in the progression of pathological cardiac remodeling and heart failure, and inhibition of RAS has become an effective means to improve cardiac remodeling in clinical practice [8]. These studies indicated that RAS plays a vital role in obesity-related cardiac dysfunction, whereas the underlying mechanism remains unclear. In this study, we demonstrated that HFD intake stimulated an excessive increase in Ang II, resulting in cardiac dysfunction. Furthermore, we found that *AT1aR* gene knockout enhanced the mitochondrial energy supply by promoting glucose and fatty acid oxidation pathways, and improved obesity-induced cardiac dysfunction, providing a new clue for the clinical treatment of related diseases.

Ang II exerts cardiovascular effects mainly by stimulating AT1R [29]. AT1R is subdivided into AT1aR and AT1bR subtypes in murine species [10]. AT1aR, which has the most homology with humans, is the most important subtype in regulating the function of the cardiovascular system [ 12, 29]. Therefore, in this study, we constructed *AT1aR* gene knockout rats to clarify the relationship between RAS and obesity-induced cardiac dysfunction. AT2R is generally considered to be a counterbalance to AT1R signaling [30]. There was no significant difference in the expression of AT2R between WT and AT1aR ^‒/‒^ rats, suggesting that *AT1aR* gene knockout did not alter the function of AT2R in myocardial tissue.

Glucose intolerance and dyslipidemia are adverse consequences of HFD, which are the causes leading to the deterioration of cardiac function [ 2, 15] and are often related to the activation of RAS [31]. These factors contributing to cardiac injury are impaired in substrate metabolism, mitochondrial dysfunction and oxidative stress [18]. Herein, we found that *AT1aR* deficiency could ameliorate insulin resistance and dyslipidemia, subsequently promoting glucose metabolism and decreasing fasting blood glucose level. These results are consistent with our previous findings [32]. Previous studies have shown that HDL could regulate the AT1R and Ang II signaling pathways, and the increase in HDL is associated with the decrease in AT1R expression [33]. The downregulation of AT1R was considered to be a novel vascular-protective effect of HDL, which has been demonstrated *in vivo* and *in vitro* in human aortic endothelial cells [34]. However, our results showed that the level of HDL was decreased in AT1aR-deficient rats, which are not consistent with the findings of previous research. Many factors, including posttranslational modifications of proteins or changes in lipids and other molecules, can affect HDL, which may lead to differences [35]. In the current study, the internal signaling mechanism of AT1aR and HDL in obesity-induced lipid metabolism disorders was still unclear, and the mechanism needs to be further studied.

The pathogenesis of hypertension in obese rats is a driving factor for the development of cardiac dysfunction. Under normal conditions, blood pressure homeostasis is regulated by a variety of mechanisms, whereas Ang II plays a pivotal role in the regulation of blood pressure only after RAS is overactivated [7]. Therefore, it can be reasonably explained that there was no significant change in the blood pressure between WT rats and AT1aR ^‒/‒^ rats fed with ND. Then, *AT1aR* deficiency attenuated HFD-induced hypertension.

In order to maintain normal systolic and diastolic functions, the myocardium requires a continuous supply of ATP [15]. Energy deficits may promote myocardial injury and contribute to cardiac dysfunction [18]. The results confirmed that *AT1aR* knockout reversed the decreasing trend in ATP level after HFD feeding. In obese rats, the decrease in energy production is caused by a variety of factors, including impairment of mitochondrial oxidative metabolism and alterations of energy substrates [ 15, 36] .

On one hand, glucose oxidation is inhibited and utilization is reduced in obese hearts [37]. Our results suggested that the gene expressions of key glycolytic enzymes, *HK2*, *PFK1* and *PK*, decreased obviously in WT rats fed with HFD, reflecting impaired glucose utilization, and *AT1aR* deficiency significantly improved glucose utilization, which indicated that AT1aR deletion enhanced the energy supply of hearts by promoting glucose oxidation. On the other hand, increased intake of fatty acids and decreased actual oxidation will also lead to an imbalance in energy supply [ 36, 38]. Transcription of genes involved in key enzymes of fatty acid β-oxidation is regulated by members of the nuclear receptor superfamily, particularly PPARs and PGC-1α [ 23 , 39]. PPARα controls enzymes involved in cellular and mitochondrial fatty acid uptake and oxidation, including FAT/CD36, malonyl CoA decarboxylase (MCD), CPT1 and long-chain acyl CoA dehydrogenase (LCAD) [15]. The transcriptional activity of PPARs is also regulated by the coactivator PGC-1α [40]. Obese rats have increased cardiac fatty acid intake, and this effect is associated with high FAT/CD36 concentrations in the sarcolemma [ 15, 41]. Mitochondrial fatty acid uptake is dependent on CPT1 which is a key enzyme that catalyzes the conversion of fatty acyl CoA into long-chain acylcarnitine in the mitochondria [ 42 , 43]. Our results confirmed that fatty acid intake was increased after HFD feeding, but *AT1aR* knockout reduced the level of free fatty acids and improved the energy supply in the heart by enhancing mitochondrial fatty acid oxidation, thus ameliorating the cardiac dysfunction caused by obesity to a certain extent.

Mitochondria are essential for the maintenance of normal cardiac function. As dynamic organelles, the morphology and function of mitochondria are closely related to each other. They are controlled by mitochondrial fusion and fission proteins, namely, mitochondrial dynamics [44]. Modifications of mitochondrial dynamics in the heart involved in energy metabolism are associated with cardiomyocyte hypertrophy, myocardial ischemia-reperfusion injury, heart failure and other cardiovascular diseases [ 25, 44]. The evaluation of mitochondrial respiratory chain complex enzyme activity is of great significance for the study of mitochondrial function under disease conditions [45]. Mitochondrial respiratory chain complex V is a key enzyme in the process of mitochondrial oxidative phosphorylation, which affects the production of ATP in the electron transport chain. In addition, lipotoxicity caused by excess fatty acid deposition in cardiomyocytes also leads to mitochondrial dysfunction, reduced energy production and insufficient energy supply to the heart [46]. In this study, the expressions of MFN2, DRP1 and FIS1 proteins supported that AT1aR deletion may promote energy supply to meet cardiometabolic requirements by affecting mitochondrial dynamics and oxidative phosphorylation.

In summary, this study suggested that RAS plays a special role in obesity-related cardiac dysfunction. *AT1aR* knockout ameliorates cardiac dysfunction by enhancing oxidative utilization of glucose and fatty acids, promoting changes in mitochondrial dynamics-related proteins and affecting cardiac energy supply. These findings revealed a new mechanism underlying the role of RAS in glucose and lipid metabolism and provided new theoretical support for the clinical application of AT1R blockers in the treatment of obesity-related cardiovascular diseases.

## Supplementary Data

Supplementary data is available at *Acta Biochimica et Biophysica Sinica* online.

## Supporting information

Supplementary_Figure_S1
