# Supplementary material for: Angiotensin II type 1a receptor knockout ameliorates high-fat diet-induced cardiac dysfunction by regulating glucose and lipid metabolism: AT1aR ‒/‒ ameliorates high-fat diet-induced cardiac dysfunction
Source: Acta Biochim Biophys Sin (Shanghai). 2023 Jul 31;55(9):1380–92. doi: 10.3724/abbs.2023054 (PMC10520472; doi:10.3724/abbs.2023054)
Supplement: Supplementary_Figure_S1 [file Supplementary_Figure_S1.pdf]

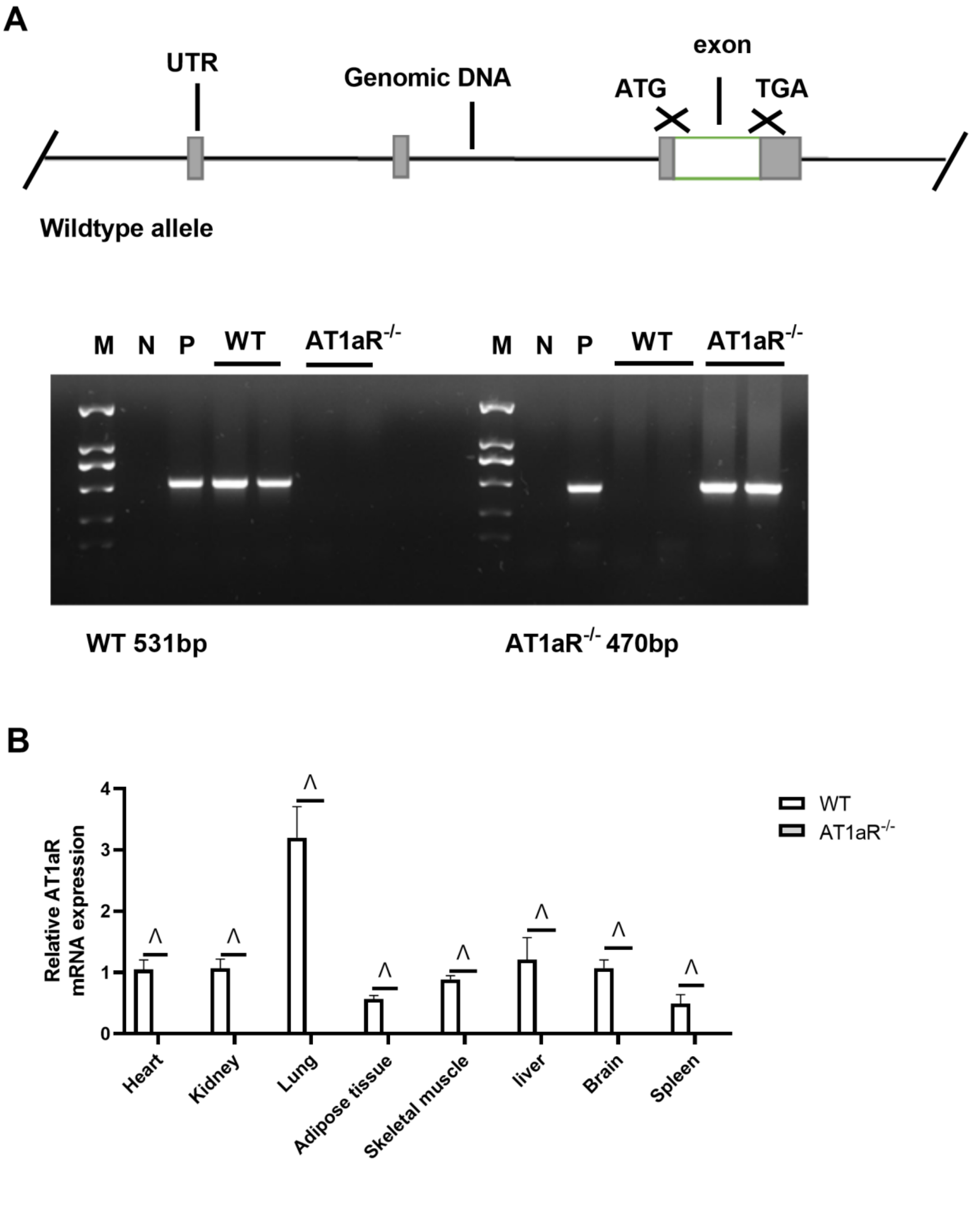

Supplementary Figure S1. Construction and gene identification of AT1aR$^{-/-}$ rats

(A) AT1aR$^{-/-}$ rats were generated by sgRNA combined with CRISPR/Cas9 system. The genes of AT1aR$^{-/-}$ and wild-type (WT) rats were identified by PCR. (B) *AT1aR* knockout in major Ang II tissues was detected by RT-PCR. Data are shown as the mean ± SEM, n=5. $^{\wedge}P < 0.05$, WT vs AT1aR$^{-/-}$ rats. M: marker; N: negative control; P: positive control.
